# The Measurement of Arterial Blood Pressure

**Published:** 1899-02-11

**Authors:** 


### THE MEASUREMENT OF ARTERIAL BLOOD PRESSURE.

IN a lecture on the Pulse and Blood Pressure, Dr. George Oliver,[1] amid much that is interesting, insists on the importance of being able, with some degree of accuracy. to estimate the blood pressure in the arteries, or, in other words, the resistance against which the heart is working. This is, he says, "the central fact of all clinically useful observation of the pulse and circulation," yet an extended experience of instrumental methods has taught him that digital observation affords but a vague and undefined impression of the blood pressure. He therefore urges the use of his homodynamometer, by the use of which the mean pressure of the blood in the artery can be approximately ascertained. This instrument consists of two parts, the pad and the recorder. The pad is a small cylindrical-shaped bag of thin rubber containing fluid. The recorder is really a small pressure gauge standardised in millimetres of mercury from the mercurial manometer, and provided with an indicator, working like the hand of a watch on a graduated dial. Placing the pad upon the artery and pressing the recorder against it, the hand of the latter moves round the face of the dial as the pressure is increased. So soon as the pressure on the pad exceeds the minimum blood pressure in the artery the indicator begins to beat with the heart. Continue the pressure and the amplitude of its beat increases; continue it still further and its amplitude diminishes again, for when the pressure on the pad is greater than the blood pressure it is only when the latter is at its heaviest that the indicator is moved. The needle then makes the widest excursion when the pressure on the bag is the same as that in the artery. The latter, therefore, is easily ascertained by noting the point at which the needle moves most definitely and widely in correspondence with the pulse. "You read the mean arterial pressure when the motion of the indicator is greatest, and in doing so you take the point midway between the two limits of the oscillation." It is a most simple affair. By means of the same instrument it is possible also to ascertain the venous pressure by applying it to a superficial vein, and noting the pressure which is required to stop the circulation through it, and this venous pressure is sometimes of some clinical importance. For example, a high arterial pressure generally maintains the normal or even a somewhat heightened venous pressure. In other words, by dint of an increased arterial tension the peripheral obstruction is overcome. Thus, as clinical observation has abundantly taught, a high tension pulse is compatible with full mental and bodily activity, and it is when the heart fails to keep up this tension that the end creeps on. But failure may also occur because the peripheral obstruction goes on increasing beyond the power of even the greatest arterial pressure to overcome it. Thus, as Dr. Oliver points out, the arterial tension may be very high, while the venous pressure is very low —as low, for example, as 10 or 5 mm., or it may not even exceed zero. In such cases, of course, the peripheral resistance must be enormous, and it is of interest to note that such a condition may be quite temporary and of nervous origin. Generally, however, increased blood pressure in the arteries means permanent peripheral obstruction, and Dr. Oliver maintains that it is a great advantage to be able to measure with some degree of accuracy the amount of effort that the heart is making to overcome it, and the amount of success which it achieves in so doing.

[1] Clin. Jour., Feb. 1.

